# Functional capacity and assistance from the caregiver during daily activities in Brazilian children with cerebral palsy

**DOI:** 10.1186/1755-7682-6-1

**Published:** 2013-01-10

**Authors:** Silvia RP Malheiros, Carlos B de Mello Monteiro, Talita Dias da Silva, Camila Torriani-Pasin, Michele SR de Andrade, Vitor E Valenti, Rodrigo Daminello Raimundo, Anelise Roosch, Luciano MR Rodrigues, Katia Valeria Manhabusque, Regina Céliac Trindade Camargo, Jefferson Drezzet, Virginia Helena Quadrado, Luiz Carlos de Abreu

**Affiliations:** 1 Faculdade Metropolitanas Unidas (FMU), Av. Santo Amaro 1239. 04506-000, São Paulo, SP, Brazil; 2 Escola de Artes, Ciência e Humanidades da Universidade de São Paulo (USP), Rua Arlindo Béttio, 1000. 03828-000, São Paulo, SP, Brazil; 3 Laboratório de Escrita Científica, Faculdade de Medicina do ABC, Av. Príncipe de Gales, 821. 09060-650, Santo André, SP, Brazil; 4 Departamento de Fonoaudiologia, Faculdade de Filosofia e Ciências, Universidade Estadual Paulista, UNESP, Av. Hygino Muzzi Filho, 737, 17525-900, Marília, SP, Brazil; 5 Departamento de Saúde Materno-infantil, Faculdade de Saúde Pública, USP, Av. Dr. arnaldo, 715. 01246-904, São Paulo, SP, Brazil

**Keywords:** Cerebral palsy, Activities of daily living, Self care

## Abstract

**Background:**

Cerebral Palsy (CP) presents changes in posture and movement as a core characteristic, which requires multiprofessional clinical treatments during children’s habilitation or rehabilitation. Besides clinical treatment, it is fundamental that professionals use evaluation systems to quantify the difficulties presented to the individual and their families in their daily lives. We aimed to investigate the functional capacity of individuals with CP and the amount of assistance required by the caregiver in day-to-day activities.

**Methods:**

Twenty patients with CP, six-year-old on average, were evaluated. The Pediatric Evaluation Inventory of Incapacities was used (PEDI - Pediatric Evaluation Disability Inventory), a system adapted for Brazil that evaluates child's dysfunction in three 3 dimensions: self-care, mobility and social function. To compare the three areas, repeated measures analysis of variance (ANOVA) were used.

**Results:**

We found the following results regarding the functional capacity of children: self-care, 27.4%, ±17.5; mobility, 25.8%, ±33.3 and social function, 36.3%, ±27.7. The results of the demand of aid from the caregiver according to each dimension were: self-care, 9.7%, ±19.9; mobility, 14.1%, ± 20.9 and social function, 19.8%, ±26.1.

**Conclusion:**

We indicated that there was no difference between the performance of the subjects in areas of self-care, mobility and social function considering the functional skills and assistance required by the caregiver.

## Background

Cerebral palsy (CP) may be defined as a disorder of posture and movement [1,2]. This disorder is persistent, however, not unalterable, and is caused by lesion in the developing central nervous system (CNS) before, during birth or in the first months of infancy [3]. Physical disability, limitations on activities and disturbances which affect sensory, perception, cognition, communication and behavior, in addition to secondary musculoskeletal problems [4] may be part of the characteristics of CP [5].

CP is the most common cause of mobility restrictions for daily living activities (DLAs) in infancy [6], with an incidence estimated at 2 cases per 1000 live births [7]. Prematurity is among the leading causes (40-50% of cases), followed by abnormal intrauterine development due to maternal-fetal infections, asphyxia, cerebral trauma during childbirth and complications in the perinatal period [7,8].

Because of sensorimotor alterations, the work of rehabilitation for individuals with CP needs to be done with the greatest possible in-depth coverage and be provided by an experienced multidisciplinary team [9-11]. O'Shea [12] cites the scope and adequate management of a multidisciplinary team is an important factor for the success of the rehabilitation program. According to Tsai et al. [9], parents should be accompanied by a multidisciplinary team that can adequately inform, as soon as possible, about the difficulties that their children will encounter in the future, considering the treatment possibilities and resources available and the potential functional results according to prognostic and predictive factors. Brasileiro et al. [13] present data that demonstrate the satisfaction of the parents of individuals with CP to be accompanied by a multidisciplinary team of health professionals, as well as being quantitatively informed about the difficulties and the real situation of the individual.

Hence even before the start of care, rehabilitation professionals should conduct assessments that allow them to know better their patients and identify the real changes in their daily activities [14,15]. By being able to identify the changes that occur in the life of families and individuals with CP, it becomes possible to understand the daily difficulties encountered, that enables a more effective therapeutic program by offering a more adequate and reliable prognosis. Furthermore, from the systems of quantification of the child's functional skills, it is possible to show the parents with more accuracy the changes presented by the patient and the justifications of the therapeutic program chosen.

Studies should be performed to allow understanding and surveying the difficulties of individuals with CP in order to improve the quality of life in subjects with this disorder. Such information could contribute to a better understanding of the requirements of the CP patient and his/her parents. Moreover, caregivers and rehabilitation professionals would also be helped for targeting the organization towards realistic objectives and proposals for interventions based on reliable data. This cross-sectional study aimed to investigate the level of dependence and need for assistance presented by individuals with CP, considering different functional areas, levels of motor impairment and need of caregiver's assistance.

## Method

This is a cross-sectional study, legally authorized representative of the patient gave written informed consent, with the study approved by the research ethics committee of the School of Medicine of the University of Sao Paulo (13364891/CAAE - 0001.0.254.186-08).

Twenty individuals with CP (11 females, 9 males) were evaluated with the mean age of 6 years and two months. Table 1 illustrates the demographic characteristics of the group considering the topographical distribution of motor change and the level of gross motor function according to the Gross Motor Function Classification System (GMFCS) [16], developed by Palisano et al. (1997) [17] and translated and adapted to the Brazilian culture by Hiratuka et al. (2010) [18] and used in different studies with cerebral palsy [19-21], with the aim to classify individuals with CP on five levels according to motor function. We excluded five CP patients that were not able to perform the study’s procedures.

**Table 1:** Group clinical characteristics of individuals with cerebral palsy

**Topographical distribution**	**GMFCS**	**Individuals**
**Diparesis**	III	6
**Hemiparesis**	II	4
**Quadriparesis**	IV	5
**Quadriparesis**	V	5
**Total**		20

Legend: GMFCS - Gross Motor Function Classification System: Level I - Walks without Limitations; Level II - Walks with Limitations; Level III - Walks Using a Hand-Held Mobility Device; Level IV - Self-Mobility with Limitations; May Use Powered Mobility; Level V - Transported in a Manual Wheelchair.

We considered the following degrees: Level I - Walks without Limitations; Level II - Walks with Limitations; Level III - Walks Using a Hand-Held Mobility Device; Level IV - Self-Mobility with Limitations; May Use Powered Mobility; Level V - Transported in a Manual Wheelchair. All children had the support of a multidisciplinary team and we took care to homogenize the population.

To quantitatively characterize the functional capacity of the individuals assessed, the Pediatric Evaluation Disability Inventory (PEDI) was used, an assessment instrument cross-culturally adapted and validated in Brazil [22] that evaluates child's dysfunction in three dimensions of self-care, mobility and social function.

In the analysis of functional abilities, each area contains a set of activities in which points are earned by individuals who are able (1 point) or unable (0 points) to accomplish specific tasks. For each skill set, we calculated the raw scores that correspond to the sum of the responses of all items assessed. As the original scores have varying scales, in order to facilitate interpretation, the scores were transformed into a scale from 0 to 100 and presented in percentage.

Within each area, caregiver assistance was also analyzed. To do so, the degree of dependence was assessed in each subset of activities, classified as: 0 = Total, 1 = Maximum, 2 = Moderate, 3 = Minimal, 4 = Supervision and 5 = Independent. Similar to the scores of functional ability, the points were added resulting in a score that was then transformed into a scale from 0 to 100 and presented as percentage. Sample size calculation provided a minimal number of 15 subjects. In the descriptive analysis, data were expressed as means, standard deviations and minimum and maximum values. To compare the three areas, repeated measures analysis of variance (ANOVA) was used. The statistical program SPSS was used and the level of significance adopted was 0.05.

## Results

The results are presented according to each area of the PEDI (self-care, mobility and social function). Table 2 is the subareas of the PEDI related to self-care, presented in order of increasing difficulty. It is important to emphasize that, to identify the subarea in which there is greater ease of performance, the mean of the results of the individuals assessed was considered.

**Table 2:** Subarea items (self-care domain) in order of increasing difficulty

	**Mean**	**SD ±**	**Min.**	**Max.**
Food textures (%)	92.5	14.3	50	100
Nose care (%)	36.0	28.7	0	100
Management of bowel (%)	34.0	34.4	0	80
Toothbrushing (%)	32.0	20.9	0	60
Management of Bladder (%)	30.0	35.8	0	100
Use of drinking containers (%)	29.0	35.2	0	100
Use of utensils (%)	28.0	27.1	0	80
Handwashing (%)	27.0	33.3	0	100
Pullover/front-opening garments (%)	24.0	27.2	0	100
Pants (%)	21.0	21.0	0	60
Hairbrushing (%)	21.0	17.7	0	60
Washing body and face (%)	15.0	19.3	0	60
Shoes/socks (%)	11.0	15.2	0	40
Toileting tasks (%)	9.0	22.9	0	80
Fasteners (%)	9.0	15.2	0	40

Legend: Results are presented in percentage.

Table 3 shows the subareas of PEDI related to mobility, in order of increasing difficulty. Thus, to identify the area in which there is greater ease of performance, the mean of each subarea of mobility was considered.

**Table 3:** Subarea items (mobility domain) in order of increasing difficulty

	**Mean**	**SD ±**	**Min.**	**Max.**
Indoor locomotion methods (%)	41.7	37.3	0	100
Chair/wheelchair transfers (%)	40.0	34.9	0	100
Indoor locomotion - pulls/carries objects (%)	37.0	40.1	0	100
Toilet transfer (%)	31.0	30.8	0	100
Bed mobility/transfers (%)	27.5	39.7	0	100
Indoor locomotion - distance/speed (%)	27.0	33.3	0	100
Outdoor locomotion - distance/speed (%)	27.0	44.1	0	100
Outdoor locomotion methods (%)	22.5	41.3	0	100
Tub transfers (%)	22.0	37.8	0	100
Outdoor locomotion - surfaces (%)	20.0	41.0	0	100
Upstairs (%)	17.0	36.9	0	100
Downstairs (%)	16.0	34.7	0	100
Car transfers (%)	11.0	26.3	0	100

Legend: Results are presented in percentage.

Table 4 presents the subareas of the PEDI related to social function, presented in order of increasing difficulty. As in other areas, the mean of each subarea of social function was considered.

**Table 4:** Subarea items (social function domain) in order of increasing difficulty

	**Mean**	**SD ±**	**Min.**	**Max.**
Comprehension of word meanings (%)	70.0	31.5	0	100
Peer interactions (%)	60.0	31.8	0	100
Complexity of expressive communication (%)	53.0	39.1	0	100
Functional use of communication (%)	52.0	41.8	0	100
Comprehension of sentence complexity (%)	48.0	41.8	0	100
Social interactive play (adults) (%)	44.0	27.2	20	100
Time orientation (%)	31.0	34.6	0	100
Play with objects (%)	30.0	35.2	0	100
Problem resolution (%)	27.0	36.9	0	100
Self information (%)	21.0	31.4	0	100
Self protection (%)	19.0	27.9	0	80
Household chores (%)	15.0	27.4	0	100
Community function (%)	12.0	22.8	0	80

Legend: Results are presented in percentage.

The following are the comparisons between areas of the PEDI, namely: self-care, mobility and social function. Thus, the data shown are divided into two parts related to the objective of the study: functional ability of individuals and caregiver assistance.

In relation to functional ability, Table 5 shows the comparison according to transformed scores, observing that social function displays a higher rate (36.3 ± 27.7). There was no statistically significant difference in the comparison between the three areas of the PEDI considering functional skills (p=0.123) or caregivers assistance (p=0,127).

**Table 5:** Comparison between areas of the PEDI, considering functional ability and caregivers assistance

**Functional skills**	**Mean**	**SD ±**	**Min.**	**Max.**
Self-care	27.4	17.5	5.5	63.0
Mobility	25.8	33.3	0	96.6
Social function	36.3	27.7	3.1	93.9
**Caregivers assistance**	**Mean**	**SD ±**	**Min.**	**Max.**
Self-care	9.7	19.9	0	70.0
Mobility	14.1	20.9	0	65.7
Social function	19.8	26.1	0	84.0

**Figure 1:**
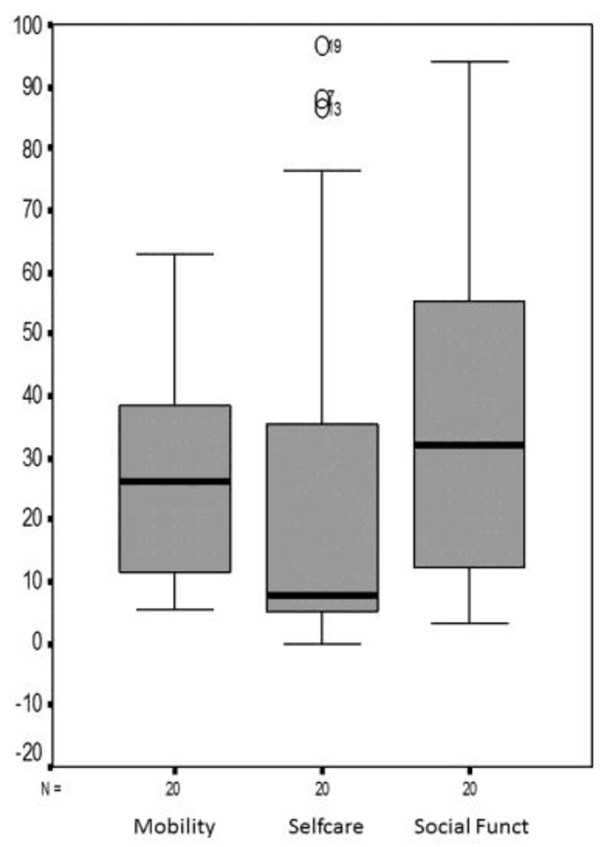
Presentation of functional ability variations.

Figure 1 shows the distribution of values through their medians, quartiles (1^st^ and 3^rd^) and minimum and maximum values (functional ability variations).

**Figure 2:**
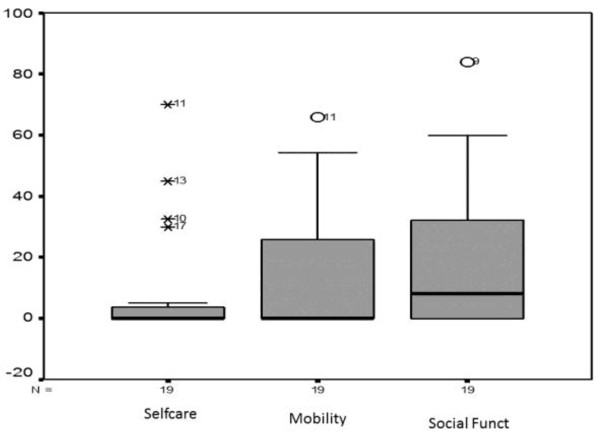
Presentation of caregiver assistance variations.

Figure 2 illustrates the distribution of values through their medians, quartiles (1^st^ and 3^rd^) and minimum and maximum values (caregiver assistance variations).

## Discussion

The objective of this study was to investigate the functional abilities of individuals with CP, as well as the level of caregiver assistance required during daily activities. The results showed that the individuals assessed had much difficulty in the areas of self-care, mobility and social function, with social function showing greater functionality.

There was no difference in performance between the subjects' functional skills and caregiver assistance required. It may have occurred because there are a large number of individuals in the group evaluated with quadriparesis and gross motor function levels IV and V, which resulted in a lower score in all areas of the PEDI.

Sorsdahl et al. [23] using the PEDI, evaluated 22 children with CP (mean age of five years and six months old) and showed mean values superior to this study in all areas, as well as higher scores in the area of mobility, both in functional skills and caregiver assistance. However, individuals participating in the study were mostly diparetic and Level I, II and III of the GMFCS, which facilitates the performance of functional tasks.

Allegretti et al. [24] used the PEDI to investigate 10 children with diparetic spastic CP, aged between 4 and 5 years old, who were compared with 10 children without any change in the same age group. The results showed significant differences between the areas of self-care and mobility. Social function did not present significant diffe-rence (p=0.132). These data corroborate the study by Oliveira and Cordani [25], who evaluated children with diparetic spastic CP and also detected worse performance in mobility and better performance in social function.

According to the results, it was found that in the area of self-care, the items with greater capacity were in the group *Food Textures,* in which all individuals performed at least half of the tasks assessed. The subarea of items with lower capacity was in *Toileting Tasks* and *Fasteners*, with no individual able to perform half of the items in these task groups as measured by the PEDI. According to Marinho et al. [26], using the PEDI in the characterization of individuals with CP, found that even with diparetic and hemiparetic individuals with increased motor function, these individuals had great difficulty in the tasks of dressing, especially in tying shoes.

It is possible to observe that these items have a greater demand for physical ability and dexterity, and the indi-viduals with CP who composed this sample were not functional enough to perform them. Domellf et al. [27] conducted studies with goal-directed reaching movements in children with CP and found that individuals with more severe motor impairment had time to perform a greater functional movement, broader patterns of movement and more segmentation with decreased function, confirming the findings of this study.

Mackenzie et al. [28] reported that the motive is not clear why individuals with CP have the inability to complete a motor task, and may be the result of problems in motor function, difficulty in understanding the task, visual deficits, or other non-motor difficulty that influences function of the individual.

Gorter et al. [29], who assessed the relationship between spasticity and gross motor function of children with 18 months old, showed that the non-affected children presented no difficulties, however, the relationship between spasticity and gross motor function decreased when including other characteristics such as muscle weakness, sensi-tivity disturbances, perception, cognition and the child's environment, which the ultimate may have influence on gross motor function.

Mancini et al. [30] performed a study that compared 142 children without neurological alterations with 33 children with CP of varying degrees of motor impairment. Were considered only 22 self-care items of the PEDI, which presented increasing degrees of difficulty, and it was found that the most difficult tasks for children without motor alteration was also the most difficult for the group with CP. These data suggest that, despite changes in individuals with CP, there are similarities in the performance of functional activities when compared to normally developing children.

In the area of mobility, the group with the highest score was *Indoor Locomotion Methods* probably because of the security offered by a controlled environment, allowing greater functionality to individuals, while the individuals assessed had the greatest difficulties in the corresponding items *Downstairs* and *Car Transfers*.

Smits et al. [31] assessed 110 children using the PEDI only in the area of mobility and observed means superior to those found in our study. Once more, the subjects assessed had better gross motor function and the study did not have participants at level V of the GMFCS. Another study that assessed the functional skills in CP was from Herrero and Monteiro [32], who demonstrated the greatest difficulty for patients with CP was in the area of mobility, and thus, the need for greater assistance from the caregiver in movements and postures that facilitate locomotion and transfer.

According to Smits et al. [31], gross motor capacity is the ability to use large muscle groups to maintain balance and perform postural changes (e.g., rolling over, sitting and walking). As CP is a major cause of motor dysfunction in children, to assess this functional loss is key to the organization of an effective therapeutic program. The authors also show that motor capacity is generally not only related to mobility and suggest that perceptual, cognitive, behavioral and communication deficits also have a great influence on the acquisition of motor skills.

In social function, although there was no statistical difference, it was where higher mean values were observed when compared to self-care and mobility in both functional skills and caregiver assistance required. This likely occurred because they are tasks that have greater cognitive demand than motor, the latter being the most difficult for patients with quadriparesis, which makes up the majority of patients assessed in our study.

The items with the greatest capacity are in the group *Comprehension of Word Meanings* and with the lowest capacity are in the group *Community Function*. These data indicate that although they are able to communicate, the individuals with CP who were assessed presented poor condition in relation to daily activities. Larsson et al. [33] carried out a study on individuals with severe motor disabilities and found that the children had become more vulnerable in concentration, motivational difficulties and the existence of influence on the process of memory due to the time taken for the communication process. Cooper et al. [34] assessed that the impact on the difficulty of the individual with CP in communication and the influence on loneliness and stress that builds friendships and close relationships are central to CP with an impact on the individuals daily lives.

Camargo et al. [35] found that children with a lower level of motor impairment tend to have greater school participation. However, these children who attend mainstream schools may have difficulty in school learning, recreation and physical activity, and express their concerns and/or interact with peers. Thus, it is possible to infer that changes in motor function may generate frustration and anguish for the family, increasing emotional burden. Knowledge of factors influencing the burden on caregivers of children with a CP is more data to be added in the planning of care and intervention of these individuals.

Sorsdahl et al. [23] conducted research through the PEDI with parents of children with CP and found that in answering the questions of the PEDI for the first time, parents present uncertainties regarding the ability of their children to perform tasks, and after participation in a group with information about motor resources and the child's need for assistance, parents were more accurate in reporting the role of their children in the home environment, suggesting that an improvement in PEDI scores after an intervention can not only be given by a change in the function of children, but also by increased knowledge and observation skills of the parents.

This study presents some limitations that are worth to be mentioned. We evaluated a small population, further studies a necessary to confirm our first hypothesis. There was no electromyographical evaluation. Unfortunately, there is not such tool in our clinic. Our results strength the relevance of early rehabilitation intervention, since, earlier the treatment onset, clinical professionals at the rehabilitation are able improve the patient rehabilitation program and recognize possible changes in the patient’s daily activities.

Our study is important, since several factors influence human development [36-42]. The developmental result of newborns with pre-natal brain injuries inducing CP may be improved. We believe that investigating the areas of mobility, self-care and social function, taken in account the functional skills and assistance required by the caregiver, we find a way to contribute to treat this disorder. It is important to mention the need of other studies that provide a greater understanding of the functional abilities of individual with CP, which will provide more adequate interventions and awareness of family members and caregivers.

## Conclusions

There was no difference between the performance of the subjects in areas of self-care, mobility and social function considering the functional skills and assistance required by the caregiver.

## Competing interests

The authors declare that they have no competing interests.

## Authors’ contributions

SRPM, CBMM,TDS, CTP, MP, MSRA, LCA, KVM, VEV, RDR, AR, JD, VHQ and LCA participated in the acquisition of data and revision of the manuscript. SRPM, TDS, CBMM, VEV,KVM and LCA determined the design, interpreted the data and drafted the manuscript. All authors read and gave final approval for the version submitted for publication.
